# Sedentary Behaviour and 12 Sleep Problem Indicators among Middle-Aged and Elderly Adults in South Africa

**DOI:** 10.3390/ijerph16081422

**Published:** 2019-04-20

**Authors:** Supa Pengpid, Karl Peltzer

**Affiliations:** 1 ASEAN Institute for Health Development, Mahidol University, Salaya, Phutthamonthon, Nakhonpathom 73170, Thailand; supaprom@yahoo.com; 2 Deputy Vice Chancellor Research and Innovation Office, North West University, Potchefstroom 2531, South Africa

**Keywords:** sedentary behaviour, sleep problems, confounding factors, adults, rural South Africa

## Abstract

The aim of this investigation was to assess the association of sedentary behaviour with 12 different sleep problem indicators among rural middle-aged and elderly adults in South Africa. Cross-sectional data were analysed from the “Health and Aging in Africa: A Longitudinal Study of an INDEPTH community in South Africa” (HAALSI) baseline survey. Participants responded to a questionnaire, including sociodemographic, health, anthropometric measures, sedentary behaviour and 12 different sleep problem indicators. The sample included 4782 individuals 40 years and older (median 61 years, interquartile range = 20 years). Overall, participants engaged <4 h (55.9%), 4–<8 h (34.1%), 8 or more hours a day (9.9%) sedentary time a day. In adjusted multinomial logistic regression, 8 h of more sedentary time was associated with short and long sleep. In adjusted logistic regression analysis, high sedentary time was positively associated with snoring, gasping, breathing stops and restless sleep and negatively associated with insufficient sleep and sleep problems due to a traumatic event. In combined analysis, compared to persons with low or moderate sedentary behaviour and moderate or high physical activity, persons with high sedentary behaviour and low physical activity were more likely to have long sleep, insufficient sleep, snoring, gasping, breathing stops, and restless sleep and less likely to have sleep problems due to traumatic events. Findings show an association between sedentary behaviour and/or combined sedentary behaviour and low physical activity with seven of 12 sleep problem indicators (short sleep, long sleep, insufficient sleep, snoring, gasping, breathing stops, and restless sleep).

## 1. Introduction

Sedentary behaviour includes sitting, being in a lying position or reclining needing a very low expenditure of energy [1], and is conceived to be independent from physical inactivity [1]. In a systematic review “strong evidence of a relationship between sedentary behaviour and all-cause mortality, fatal and non-fatal cardiovascular disease, type 2 diabetes and metabolic syndrome” was found [2].

Sleep problems or insomnia is one of the most common health problems, affecting approximately 10% to 20% of the world population [3]. “The etiology and pathophysiology of insomnia involve genetic, environmental, behavioral, and physiological factors culminating in hyperarousal.” [3]. Both short and long sleep durations can increase the risk of morbidity (diabetes mellitus, cardiovascular disease, coronary heart disease, and obesity) and mortality [4,5]. Several studies [6,7,8] found an association between sedentary behaviour and short and/or long sleep. In a recent meta-analysis, sedentary behaviour increased the odds for insomnia and sleep disturbance [9]. There are limited studies investigating sedentary behaviour and sleep indicators in low- and middle-income countries. An exception is a study in six middle-income countries in predominantly older adults, where general sedentary time was overall associated with sleep problems [10]. The aim of this study was to assess the association of sedentary behaviour with 12 different sleep problem indicators among rural middle-aged and elderly adults in South Africa. Sleep problems have been previously reported to be common in South Africa, 7.1% insomnia in the general adult population, 9.6% among middle-aged (45–64 year-olds) and 20.5% among 65 years and older persons [11].

## 2. Methods

### 2.1. Sample and Procedure

Baseline data were analysed from the “Health and Ageing in Africa: A Longitudinal Study of an INDEPTH Community in South Africa (HAALSI) in the INDEPTH Health and Demographic Surveillance System (HDSS) site of Agincourt” in 2015 in rural South Africa [12]. The baseline data used in this study is publicly available at the Harvard Center for Population and Development Studies (HCPDS) program website (www.haalsi.org). Based on completed home interviews (response rate 85.9%) [12], 4782 individuals were included with complete sedentary behaviour measurements. “Signed informed consent was obtained from all respondents prior to assessments. The study received ethical approvals from the University of the Witwatersrand Human Research Ethics Committee (ref. M141159), the Harvard T.H. Chan School of Public Health, Office of Human Research Administration (ref. C13–1608–02), and the Mpumalanga Provincial Research and Ethics Committee.” [12].

### 2.2. Measures

#### 2.2.1. Exposure Variables

*Sedentary behaviour* was assessed with two items “On a usual week day, how many hours/minutes did you spend sitting or reclining (excluding sleep)? This may include time sitting on a chair or bench, visiting friends, reading, sitting in church, sitting down to watch television” on a usual weekday or weekend day [12,13]. Sedentary time was classified into <4 h, 4–<8 h, and 8 or more hours a day [14].

#### 2.2.2. Outcome Variables

*Sleep duration* was assessed with the question, “Over the past four weeks, how many hours do you think you actually slept each day?” [15,16]. Responses were divided into three categories: normal sleep (7–9 h), short sleep (≤6 h) and long sleep (≥10 h) [17].

*Bad sleep quality.* “Over the past 4 weeks, how would you rate your sleep quality overall?” (Very good/Good vs. Very bad/Bad) [15,16].

*Insufficient sleep.* “How often during the past 4 weeks did you get enough sleep to feel rested upon waking up?” (Sometimes/often/very vs. rarely/never) [15,16].

*Awakenings.* ”During the past 4 weeks, how often did you wake up in the middle of the night or early morning?” (<once per week vs. at least once per week) [15,16].

*Snoring.* “During the past month, have you snored loudly, or ever been told that you were snoring loudly?” (Yes/No) [15,16].

*Gasping.* “During the last month, have you had, or ever been told that you were snorting or gasping?” (Yes/No) [15,16].

*Breathing stops.* “During the last month, have you had, or ever been told that your breathing stops or you struggle for breath?” (Yes/No) [15,16].

*Sleep latency* (>30 min). “During the past 4 weeks, how often could you not get to sleep within 0 min?” (”never or <once a week or once or twice a week” vs. ”three or more times a week”) [15,16].

*Restless sleep.* ”Much of the time in the past week, your sleep was restless?” (Yes/No) [18].

*Pain has interfered with your sleep.* “On a scale of 0 to 10, where 0 is ‘Does not interfere’ and 10 is ‘Completely interferes’, select the one number that describes how, during the past 24 h, pain has interfered with your sleep?” (zero vs. 1–10 interferes) [19].

*Sleep problem due to traumatic event.* “In your life, have you ever had any experience that was so frightening, horrible, or upsetting that, in the past 30 days you had more trouble than usual falling asleep or staying asleep?” (Yes/No) [20].

#### 2.2.3. Confounding Variables

*Sociodemographic information* included sex, age, formal education, and a “wealth index in quintiles created from household characteristics and ownership of household items, livestock, and vehicles” [12,19].

*Social cohesion* was measured with four questions, e.g., “Most people in this village are willing to help their neighbours” Response options ranged from 1 = strongly agree to 4 = strongly disagree [12] (Cronbach’s alpha 0.87). “High social cohesion was defined as reversed scores of 13–16” [21].

*Current tobacco use* was assessed with “two questions on current smoking and current smokeless tobacco use, such as snuff, chewing tobacco, snus, and betel with tobacco” [12,21].

*Alcohol dependence* was assessed with the four-item “Cut down-Annoyed-Guilty-Eye opener” (CAGE) questionnaire [22] (Cronbach’s alpha 0.82).

*Physical activity* was assessed with the “General Physical Activity Questionnaire (GPAQ)” [23,24]. Following GPAQ guidelines [24], results were classified into “low, moderate, and high physical activity” [24].

*Body Mass Index* (BMI) was calculated from standardized measures of height and body weight [13], and classified into “underweight (<18.5 kg/m^2^), normal weight (18.5–24.9 kg/m^2^), overweight (25–29.9 kg/m^2^), and obesity (30+ kg/m^2^),” using World Health Organization (WHO) criteria [21,25].

*Self-reported health status* was measured with the item: “In general, how would you rate your health today?” [12]. “Responses were grouped into 1 = very bad or bad, 2 = moderate, and 3 = good or very good.” [21].

## 3. Data Analysis

Associations between sedentary behaviour and sleep duration were analysed with multinomial regression, with short (≤6 h) and long sleep (≥10 h) duration as dependent variable, and normal sleep duration (7–9 h) as reference category. Logistic regression was used to estimate the associations between sedentary behaviour and other sleep problem indicators. All models were adjusted for sex, age, education, wealth status, social cohesion, BMI, current tobacco use, problem drinking, and self-rated health status. Moreover, we used multivariable multinomial and logistic regression to estimate the combined relationship between sedentary behaviour and physical activity with problem sleep indicators. For the combined multinomial and logistic regression analysis, the sample was sub-divided based on sedentary and physical activity levels into four groups: (1) low or moderate sedentary time (<8 h) plus moderate or high physical activity group (reference category), (2) low or moderate sedentary time (<8 h) plus low physical activity group, (3) High sedentary time (≥8 h) plus moderate or physical activity group, and (4) high sedentary time (≥8 h) plus low physical activity. *p* < 0.05 was considered significant. All statistical procedures were conducted with STATA software version 14 (Stata Corporation, College Station, TX, USA).

## 4. Results

### 4.1. Sample Characteristics

The sample included 4782 individuals (40 years and older, median 61 years, interquartile range = 20 years), 53.3% were female, 93.2% had moderate to high social cohesion, 58.1% overweight or obese, and 42.1% engaged in low physical activity. Overall, 68.8% of respondents reported good self-rated health status, 1.4% alcohol dependence, and 15.6% were current tobacco users.

Overall, participants engaged <4 h (55.9%), 4–<8 h (34.1%), and 11 or more hours a day (9.9%) sedentary time a day. Of all participants, 13.1% had short sleep (≤6 h) and 18.2% long sleep (≥10 h). Other sleep problem indicators included, awakening (40.0%), snoring (19.6%), gasping (12.7%), breathing stops (7.1%), bad sleep quality (6.3%), sleep latency (9.0%), restless sleep (32.1%), pain that interferes with sleep (9.5%), problems with sleep due to traumatic experience (15.0%) and insufficient sleep (14.4%) (see Table 1).

### 4.2. Associations of Sedentary Behaviour with Sleep Indicators

In adjusted multinomial logistic regression analysis, 8 h of more sedentary time was associated with short sleep and long sleep. In adjusted logistic regression analysis, 8 h of more sedentary time was associated with snoring, gasping, breathing stops and restless sleep, and 4–<8 h sedentary behaviour was positively associated with snoring and negatively associated with bad sleep quality, breathing stops and restless sleep. Four or more hours sedentary time was negatively associated with insufficient sleep and sleep problems because of a traumatic event. No associations were found between sedentary behaviour and latency, nocturnal awakenings, and pain that interferes with sleep (see Table 2).

### 4.3. Associations of Combined Sedentary Behaviour and Physical Inactivity with Sleep Problem Indicators

In adjusted multinomial logistic regression analysis, compared to persons with low or moderate sedentary behaviour and moderate and high physical activity, persons with high sedentary behaviour and moderate or high physical activity were more likely to have short sleep (Relative Risk Ratio—RRR: 2.06, Confidence Interval—CI: 1.21, 3.52), and persons with low or moderate sedentary behaviour and low physical activity (RRR: 1.32, CI: 1.11, 1.57) as well as persons with high sedentary behaviour and low physical activity (RRR: 1.80, CI: 1.23, 2.63) were more likely to have long sleep. In adjusted logistic regression analysis, compared to persons with low or moderate sedentary behaviour and moderate or high physical activity, persons with high sedentary behaviour and low physical activity were more likely to have snoring (Odds Ratio—OR: 1.51, CI: 1.07, 2.13), gasping (OR: 1.88, CI: 1.29, 2.72), breathing stops (OR: 1.67, CI: 1.13, 2.41), restless sleep (OR: 1.56, CI: 1.13, 2.17) and insufficient sleep (OR: 1.62, CI: 1.02, 2.57) and less likely to have a sleep problem due to a traumatic event (OR: 0.41, CI: 0.22, 0.75). In addition, compared to persons with low or moderate sedentary behaviour and moderate and high physical activity, persons with low or moderate sedentary behaviour and low physical activity were more likely to have bad sleep quality (OR: 1.55, CI: 1.17, 2.05), latency (OR: 1.25, CI: 1.09, 1.43), nocturnal awakenings (OR: 1.17, CI: 1.02, 1.33), insufficient sleep (OR: 2.89, CI: 2.39, 3.49) and pain interfering with sleep (OR: 2.04, CI: 1.61, 2.57), and were less likely to have snoring (OR: 0.79, CI: 0.67, 0.94) and gasping (OR: 0.74, CI: 0.60, 0.91). Further, persons with high sedentary behaviour and moderate or high physical activity were less likely to have nocturnal awakenings (OR: 0.35, CI: 0.20, 0.60) and more likely to have insufficient sleep (OR: 2.53, CI: 1.45, 4.41) and restless sleep (OR: 1.57, CI: 1.01, 2.45) (see Table 3).

## 5. Discussion

This is one of the first studies to investigate the association of sedentary behaviour with 12 different sleep problem indicators in a large sample in South Africa. Results found that high sedentary time (≥8 h), adjusted for relevant confounders such as sociodemographics, body mass index and social cohesion, was associated with six sleep problem indicators: short sleep, long sleep, snoring, gasping, breathing stops, and restless sleep. In addition, a combined association of high sedentary time (≥8 h) and low physical activity was found with six sleep problem indicators: long sleep, snoring, gasping, breathing stops, insufficient and restless sleep. It should be acknowledged that someone who is awake for longer (shorter sleep time) is likely to have higher awake sedentary time just by virtue of being awake for longer. This may explain as to why in the combined analysis the combined association of high sedentary time (≥8 h) and low physical activity was no longer significant on short sleep. Stamtakis et al. [26] found that, “although replacing sedentary behaviour with walking and moderate-to-vigorous physical activity are associated with the lowest mortality risk, replacements with equal amounts of standing and sleeping (in low sleepers only) are also linked to substantial mortality risk reductions.”

Several previous studies [6,7,8] also found a positive association between sedentary behaviour and short and/or long sleep. A previous systematic review also found a positive association between sedentary behaviour and multiple sleep problems among adults [9]. Various possible mechanisms have been suggested for the link between sedentary behaviour and sleep problems, including elevated depression risk, increase in metabolic syndrome and increased LED-backlit TV exposure, all of which have a higher risk for sleep problems [9,10]. Kline et al. [27] found that “exercise training may be helpful for improving aspects of daytime functioning of adults with obstructive sleep apnea”, which may explain our association between sedentary behaviour and apnea symptoms (gasping, breathing stops). One study in six middle-income countries also found an association between general sedentary time and sleep problems [10]. More research is needed in low- and middle-income countries on the association between sedentary behaviour and different sleep problem indicators, so as to compare such findings with results from high-income countries.

Sedentary time was in this study negatively associated with insufficient sleep and sleep problems due to trauma, and no association was found with bad sleep quality, latency, nocturnal awakenings, and pain that interferes with sleep. In a previous review [9], also no association between sedentary behaviour and poor sleep quality and/or daytime sleepiness (insufficient sleep to feel rested). It is possible that higher sedentary time may include small sleeping naps, especially among older adults, so that higher sedentary time compensates for insufficient sleep. In the combined analysis, however, high sedentary behaviour and physical inactivity, was associated with insufficient sleep in this study. Sedentary time was consistently negatively associated with sleep problems due to trauma in this study. Talbot et al. [28] found that “worse sleep quality predicts lower physical activity in posttraumatic stress disorder.” It is possible that persons with higher sedentary behaviour have more opportunity to deal with their traumatic experiences during the day time, reducing sleep problems due to traumatic events.

### Study Limitations

Both sedentary and sleep behaviour were assessed by self-report, and future studies should include objective measures. Sedentary time may have been under-reported and physical activity over-reported because of social desirability bias [26]. Self-reported sleep duration may be an overestimation of the actual measured sleep duration [29]. Since the study was cross-sectional, no causal inferences can be made.

## 6. Conclusions

Findings show an association between sedentary behaviour and/or combined sedentary behaviour and low physical activity with seven of 12 sleep problem indicators (short sleep, long sleep, insufficient sleep, snoring, gasping, breathing stops, and restless sleep).

## Figures and Tables

**Table 1:** Sample characteristics by sleep problem indicators and sedentary time.

Sleep Problem Indicator	Sample	Sedentary Time		
		<4 h/day	4–<8 h/day	≥8 h/day
	*N* (%)	*N* (%)	*N* (%)	*N* (%)
Short sleep (≤6 h)	600 (13.1)	313 (12.4)	214 (13.5)	73 (15.7)
Long sleep (≥10 h)	831 (18.2)	442 (17.6)	262 (16.5)	127 (27.3)
Bad sleep quality	303 (6.3)	180 (6.7)	86 (5.3)	37 (7.8)
Insufficient sleep	690 (14.4)	507 (19.0)	121 (7.4)	62 (13.1)
Latency	429 (9.0)	232 (8.7)	165 (10.1)	32 (6.8)
Nocturnal awakenings	1910 (40.0)	1093 (40.9)	623 (38.2)	194 (41.0)
Snoring	931 (19.6)	443 (16.6)	336 (20.7)	152 (32.4)
Gasping	606 (12.7)	273 (10.2)	204 (12.5)	129 (27.4)
Breathing stops	339 (7.1)	188 (7.1)	90 (5.5)	61 (13.0)
Restless sleep	1504 (32.1)	850 (32.3)	456 (28.6)	198 (43.5)
Sleep problem due to traumatic event	702 (15.0)	484 (18.4)	181 (11.4)	37 (8.2)
Pain interfered with sleep	445 (9.5)	246 (9.4)	145 (9.1)	54 (11.9)

**Table 2:** Associations of sedentary behaviour with sleep problem indicators.

Sleep Problem Indicator	Sedentary Quartiles (Referent: <4 h/day)	
	4–<8 h/day	≥8 h/day
	ARRR (95% CI) ^1^	ARRR (95% CI) ^1^
Short sleep (≤6 h)	1.06 (0.87, 1.30)	1.69 (1.24, 2.31) ***
Long sleep (≥10 h)	0.93 (0.77, 1.11)	1.55 (1.19, 2.03) ***
	AOR (95% CI) ^1^	AOR (95% CI) ^1^
Bad sleep quality	0.70 (0.51, 0.95) *	1.05 (0.60, 1.88)
Insufficient sleep	0.34 (0.27, 0.43) ***	0.60 (0.43, 0.82) **
Latency	1.10 (0.88, 1.34)	0.66 (0.43, 1.01)
Nocturnal awakenings	0.88 (0.77, 1.01)	0.92 (0.73, 1.14)
Snoring	1.22 (1.03, 1.44) *	2.27 (1.78, 2.91) ***
Gasping	1.18 (0.96, 1.45)	3.15 (2.42, 4.11) ***
Breathing stops	0.71 (0.54, 0.95) *	1.91 (1.35, 2.70) **
Restless sleep	0.80 (0.69, 0.92) **	1.39 (1.11, 1.75) **
Sleep problem due to traumatic event	0.56 (0.46, 0.69) ***	0.33 (0.22, 0.50) ***
Pain interfered with sleep	0.93 (0.73, 1.19)	1.00 (0.69, 1.46)

*** *p* < 0.001; ** *p* < 0.01; * *p* < 0.05; ARRR = Adjusted Relative Risk Ratio; AOR = Adjusted Odds Ratio; CI = confidence interval; ^1^ Adjusted for sex, age, education, wealth status, social cohesion, BMI, current tobacco use, problem drinking, and self-rated health status.

**Table 3:** Associations of combined sedentary behaviour and physical activity with sleep problem indicators.

Sleep Problem Indicator	Sedentary Low or Moderate and Physical Activity (PA) Moderate or High (*n* = 1536) (Referent)		
	Sedentary Low/Moderate and PA Low (*n* = 1764)	Sedentary High and PA Moderate or High (*n* = 94)	Sedentary High and PA Low (*n* = 237)
	ARRR (95% CI) ^1^	ARRR (95% CI) ^1^	ARRR (95% CI) ^1^
Short sleep (≤6 h)	0.87 (0.76, 1.06)	2.06 (1.21, 3.52) ***	1.16 (0.72, 1.89)
Long sleep (≥10 h)	1.32 (1.11, 1.57) **	0.94 (0.51, 1.75)	1.80 (1.23, 2.63) **
	AOR (95% CI) ^1^	AOR (95% CI) ^1^	AOR (95% CI) ^1^
Bad sleep quality	1.55 (1.17, 2.05) **	0.95 (0.34, 2.68)	1.78 (0.97, 3.24)
Insufficient sleep	2.89 (2.39, 3.49) ***	2.53 (1.45, 4.41) ***	1.62 (1.02, 2.57) *
Latency	1.25 (1.09, 1.43) **	0.70 (0.43, 1.16)	0.75 (0.53, 1.08)
Nocturnal awakenings	1.17 (1.02, 1.33) *	0.35 (0.20, 0.60) ***	1.11 (0.80, 1.53)
Snoring	0.79 (0.67, 0.94) *	0.55 (0.29, 1.06)	1.51 (1.07, 2.13) *
Gasping	0.74 (0.60, 0.91) **	1.33 (0.73, 2.39)	1.88 (1.29, 2.72) ***
Breathing stops	1.05 (0.81, 1.37)	1.68 (0.82, 3.45)	1.67 (1.13, 2.41) **
Restless sleep	0.95 (0.82. 1.09)	1.57 (1.01, 2.45) *	1.56 (1.13, 2.17) **
Sleep problem due to traumatic event	1.04 (0.87, 1.25)	0.60 (0.29, 1.27)	0.41 (0.22, 0.75) **
Pain interfered with sleep	2.04 (1.61, 2.57) ***	1.01 (0.42, 2.40)	1.53 (0.91, 2.55)

*** *p* < 0.001; ** *p* < 0.01; * *p* < 0.05; ARRR = Adjusted Relative Risk Ratio; AOR = Adjusted Odds Ratio; CI = confidence interval; ^1^ Adjusted for sex, age, education, wealth status, social cohesion, BMI, current tobacco use, problem drinking, and self-rated health status.
